# Atypical deletion of Williams–Beuren syndrome reveals the mechanism of neurodevelopmental disorders

**DOI:** 10.1186/s12920-022-01227-7

**Published:** 2022-04-04

**Authors:** Jianrong Zhou, Ying Zheng, Guiying Liang, Xiaoli Xu, Jian Liu, Shaoxian Chen, Tongkai Ge, Pengju Wen, Yong Zhang, Xiaoqing Liu, Jian Zhuang, Yueheng Wu, Jimei Chen

**Affiliations:** 1 Department of Cardiovascular Surgery of Guangdong Cardiovascular Institute, Guangdong Provincial People’s Hospital, Guangdong Academy of Medical Sciences, Guangzhou, China; 2 Guangdong Provincial Key Laboratory of South China Structural Heart Disease, Guangdong Provincial People’s Hospital, Guangdong Academy of Medical Sciences, Guangzhou, China; 3 Department of Nutrition, Guangdong Provincial People’s Hospital, Guangdong Academy of Medical Sciences, Guangzhou, China; 4 Department of Physical Therapy and Rehabilitation, Guangdong Provincial People’s Hospital, Guangdong Academy of Medical Sciences, Guangzhou, China; 5 Department of Endocrinology, General Hospital of Central Theater Command, Wuhan, China; 6 Research Department of Medical Sciences, Guangdong Provincial People’s Hospital, Guangdong Academy of Medical Sciences, Guangzhou, China; 7 Division of Epidemiology, Guangdong Provincial People’s Hospital and Cardiovascular Institute, Guangzhou, China

**Keywords:** Atypical deletion, Williams–Beuren syndrome, Genotype–phenotype correlation, Intellectual disability, Growth restriction

## Abstract

Genes associated with specific neurocognitive phenotypes in Williams–Beuren syndrome are still controversially discussed. This study identified nine patients with atypical deletions out of 111 patients with Williams–Beuren syndrome; these deletions included seven smaller deletions and two larger deletions. One patient had normal neurodevelopment with a deletion of genes on the distal side of the Williams–Beuren syndrome chromosomal region, including *GTF2I* and *GTF2IRD1*. However, another patient retained these genes but showed neurodevelopmental abnormalities. By comparing the genotypes and phenotypes of patients with typical and atypical deletions and previous reports in the literature, we hypothesize that the *BAZ1B*, *FZD9*, and *STX1A* genes may play an important role in the neurodevelopment of patients with WBS.

**Supplementary Information:**

The online version contains supplementary material available at 10.1186/s12920-022-01227-7.

## Introduction

Williams–Beuren syndrome (WBS; OMIM number 194050), also known as Williams syndrome, is a disorder that affects multiple systems. It is caused by the heterozygous deletion of 1.55–1.84 Mb on chromosome 7q11.23, which is a fragment containing approximately 26 to 28 genes [1]. Genome rearrangement often occurs in this region because low-copy repeats are located on both sides of the common deletion region, which leads to nonallelic recombination during meiosis [2]. This region is also referred to as the Williams–Beuren syndrome chromosomal region (WBSCR). It has been estimated that the prevalence of WBS is approximately 1/7500–1/20,000 [3]. Although the phenotype features extensive heterogeneity in severity and performance, patients usually show facial dysmorphism, cardiovascular abnormalities, intellectual disability, specific cognitive characteristics, developmental limitations, hypothyroidism, infantile hypercalcemia, and other clinical symptoms that affect multiple organs and systems [4]. However, it remains unclear how these gene deletions cause the characteristic phenotype of WBS, and this uncertainty may be related to the low expression of gene products.

Most patients have the same deletion span, but few individuals have smaller or larger deletion fragments. To study the relationship between genotype and phenotype, patients with atypical deletions are promising research objects. However, due to the low incidence of WBS and rarity of the atypical deletions (only 2–5% of WBS patients) [1, 2], the number of subjects available for research is extremely limited. To date, deletion of the elastin (*ELN*) gene has been identified as the main cause of cardiovascular disorders in WBS patients, especially arterial stenosis [5]. Moreover, several researchers have used patients with atypical deletions and animal experiments to show that the heterozygous deletion of genes located on the distal side of the WBSCR (i.e., *GTF2I*, *GTF2IRD1*, and *CLIP2*) is the main reason for the behavior and cognitive phenotype of WBS patients [6, 7]. Other studies suggested that the deletion of genes on the centromere side of the WBSCR also contributes to the specific phenotype of WBS patients [8, 9].

This study describes nine cases of Chinese WBS patients with atypical deletions, one of which showed normal neurocognitive development. The clinical phenotypic characteristics and genomic imbalances of these patients were used to verify and expand the findings of previous literature. Moreover, we discuss the contributions of several gene deletions in the WBSCR to the symptoms of WBS patients. High-resolution molecular testing is recommended for WBS patients, especially those with nonclassical clinical symptoms. In this way, more comprehensive and accurate genetic information can be obtained, which can enable accurate diagnosis and treatment.

## Materials and methods

### Patient subjects

The research plan was approved by the Research Ethics Committee of Guangdong Provincial People’s Hospital [No. GDREC2019587H(R1)]. Informed written consent was obtained from the patients’ parents.

Patients who had been diagnosed with WBS or were clinically suspected to have WBS by the Lowery scoring system [10] were recruited. Their clinical data including medical records, gestational age, birth weight, birth length, echocardiography, heart catheterization findings, gene test reports, and family history were reviewed.

### Genetic testing

Chromosomal microarray analysis (CMA) was used to detect the genomic imbalance of patients with WBS. Approximately 2.0 ml of peripheral venous blood was collected from the patients and their parents. Genomic DNA was extracted using the QIAamp DNA Mini Kit (QIAGEN GmbH, Germany) according to the manufacture’s instructions.

DNA samples (250 ng) were hybridized with an Affymetrix Cytoscan 750 K array (Affymetrix, Santa Clara, CA, USA), which contains more than 750,000 markers for copy number analysis and 200,000 single nucleotide polymorphism (SNP) probes for genotyping. After hybridization, Chromosome Analysis Suite software (Affymetrix, USA) and human genome version GRCh37 (hg19) were used to analyze the results. The detected copy number variation (CNV) was compared with internal and national public CNV databases, such as the Database of Genomic Variants (DGV), the International Standards for Cytogenomic Arrays Consortium, and the Online Mendelian Inheritance in Man.

According to the latest standards and guidelines for sequence variations, as developed by the American College of Medical Genetics and Genomics [11], the CMA results were divided into five grades: “pathogenicity”, “possible pathogenicity”, “uncertain significance”, “possible benign”, and “benign”.

### Quantitative real time PCR (qPCR)

qPCR was performed on four samples (No. 6 and No. 7 atypical deletion patients, a typical deletion patient and a normal control) by quantitative analysis and the standard curve method [12]. All qPCRs were performed as previously described [13]. The PCR primer sequences used for the amplification of microsatellite markers were selected from the USCS database between positions 71,449,000 to 73,925,000 on chromosome 7 as previously described [12]. Twenty-eight pairs of PCR primers generated amplified fragments along the WBS deleted region (2.5 Mb) in the 100–300 kb interval. The *SOX9* gene on chromosome 17 was used as an internal reference gene. The comparative C_t_ method was used to determine the relative content for confirmation of the CMA results [14].

### Cardiovascular status assessment

Clinical data, including medical records, electrocardiograms, echocardiography, and cardiac catheterization reports were systematically reviewed. And WBS patients who voluntarily came to the center for physical examination were subjected to cardiac ultrasound examination by pediatric cardiologists. Supravalvular aortic stenosis (SVAS) was diagnosed by echocardiography when the pressure gradient (PG) exceeded 10 mm Hg. Pulmonary stenosis (PS) was diagnosed if the main or branch pulmonary artery showed local stenosis or diffuse stenosis with a PG exceeding 10 mm Hg [15]. Coarctation of the aorta (CoA) was defined by echocardiography as peak PG exceeding 40 mm Hg at the distal aortic arch [15].

### Physical development assessment

The height (accurate to 0.1 cm) and weight (accurate to 0.1 kg) of patients without shoes and in light clothes were measured by electronic height and weight meters. According to the patients’ sex, date of birth, and date of the visit, the Z scores of height-for-age (HAZ), weight-for-age (WAZ), and body mass index (BMI, i.e., the weight in kg divided by the height in m^2^)-for-age (BAZ) were calculated by WHO Anthro software (https://www.who.int/childgrowth/software/en/). Based on the WHO 2006 and 2007 growth reference standards [16], stunting was defined as HAZ < − 2, underweight was defined as WAZ < − 2, and emaciation was defined as BAZ < − 2.

### Neurodevelopmental assessment

The Gesell development schedule (GDS) is one of the commonly used methods to assess the neurological and intellectual development of infants and children in China [17, 18]. In the present study, a version of the GSD that had been adjusted by the Chinese Pediatric Association [19] was used to evaluate the neurodevelopment of WBS patients. The assessment was conducted by trained rehabilitation doctors. The assessment contents included gross motor functions, fine motor functions, adaptive behavior, language, and social behavior. Each test obtained a development quotient (DQ), and the total average DQ was obtained by calculating the average of the five DQs. A higher DQ indicates a higher level of neurodevelopment in each domain.

### Statistical analysis and presentation

SPSS Statistics software 20.0 (IBM, Armonk, NY, USA) was used for all statistical evaluations. The collected data are expressed as the mean ± SD. Figures were prepared by GraphPad Prism 8.0.1 (San Diego, CA, USA) and Adobe Illustrator CC 2019 (NY, USA).

## Results

### Deletion mapping in patients with atypical deletions

**Fig. 1:**
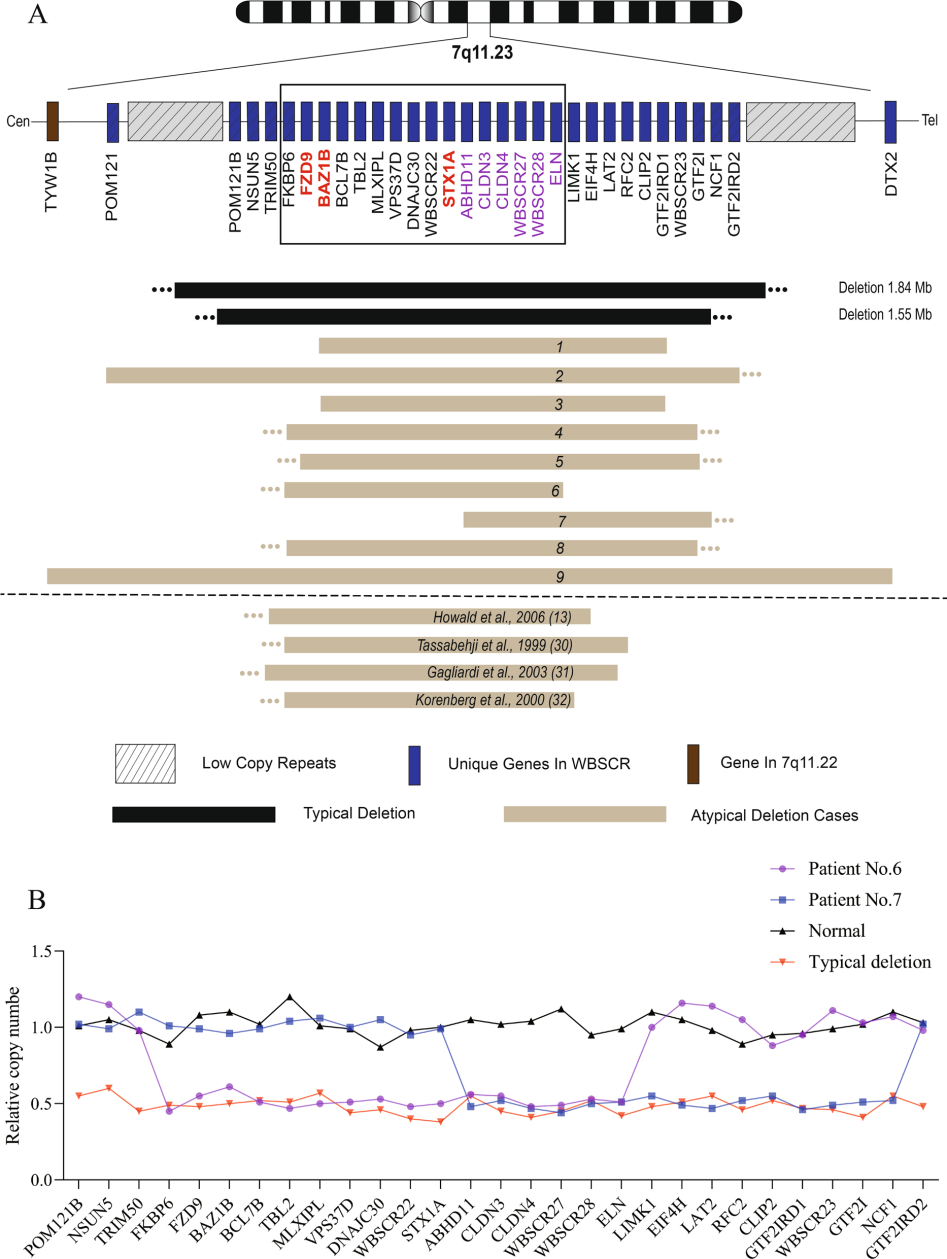
A pattern of atypical deletions detected in patients with WBS. **A** The degree of deletion in WBS patients with typical deletions is indicated by black bars; it is approximately 1.55–1.84 Mb in size. The gray bars below represent the gene deletion fragments of WBS patients with atypical deletion, including the nine patients in this cohort and four cases of previously reported deletion genes that did not include the WBSCR distal-side genes such as *GTF2I* and *GTF2IRD1*. A panel was used to highlight the deletion region that commonly overlapped between the current study and the previous cases with a panel, and the gene symbols of interest in the candidate region are marked in red. The names of deleted genes with minimal common overlap are marked in purple. **B** The CMA results were verified by qPCR, and the deletion genes and chromosome loci of patients with atypical deletions (No. 6 and No. 7) were confirmed

In this study, a total of 111 patients with WBS were recruited, including nine patients with atypical deletions. All nine cases were de novo mutations without a family history. As shown in Fig. 1A, all nine patients had heterozygous microdeletions in chromosome 7q11.23, ranging from 741 kb to 4.06 Mb, and the deletion sites differed. By searching the database, all of the above CNVs were pathogenic and related to WBS.

The chromosome deletion breakpoints of patients No. 1 and No. 3 were almost identical, and genes from *BAZ1B* to *GTF2IRD1* were deleted in both patients. The deletion of patient No. 2 extended in the proximal direction, ranging from *POM121* to *GTF2IRD2*. Although the deletion sites of patients No. 4 and No. 8 differed, the deleted genes ranged from *FKBP6* to *GTF2I*. Compared with patient No. 4, the *FKBP6* gene was not deleted in patient No. 5, but the other gene deletions were identical. Patient No. 6 had deletions from the *FKBP6* gene to the *ELN* gene, excluding the WBSCR genes on the distal side, such as *GTF2I* and *GTF2IRD1*. In contrast, patient No. 7, the gene related to the distal side of *ABHD11* was deleted, but the gene on its proximal side was retained. Genotypes of patients No.6 and No.7 were confirmed by qPCR, as shown in Fig. 1B. Moreover, the deleted fragment of patient No. 9 was the longest, and several genes in the 7q11.22 region were also deleted. No other pathogenic CNV or mutated gene was found in these patients by whole-exon sequencing.

### Characteristics of participants

Table 1 lists the WBS patients with atypical deletions and their clinical characteristics. The age of these patients (two females and seven males) ranged from 11 to 38 months (24.44 ± 9.85 months).

**Table 1:** WBS patients with atypical microdeletion in 7q11.23 and their clinical characteristics

Case no	Sex	Age (month)	Gene tests	Deletion		Growth retardation	Cardiovascular diseases	Mental disability	Inguinal hernias	Endocrine abnormalities	Typical face
				Range (hg19)	Size						
1	M	38	CMA	Del (7q11.23) (72,858,312–74,071,135) × 1	1.213 Mb	+	+	+	+	−	+
2	F	24	CMA	Del (7q11.23) (72,351,682–74,264,871) × 1	1.91 Mb	+	+	+	−	−	−
3	M	27	CMA	Del (7q11.23) (72,858,305–74,071,087) × 1	1.212 Mb	−	+	+	+	−	+
4	M	31	CMA	Del (7q11.23) (72,745,738–74,129,824) × 1	1.384 Mb	+	+	+	−	−	+
5	F	11	CMA	Del (7q11.23) (72,800,000–74,150,000) × 1	1.35 Mb	+	+	+	+	−	+
6	M	32	CMA	Del (7q11.23) (72,742,276–73,483,030) × 1	0.741 Mb	−	+	+	+	+	+
7	M	31	CMA	Del (7q11.23) (73,150,001–74,200,000) × 1	1.05 Mb	−	+	−	+	−	+
8	M	13	CMA	Del (7q11.23) (72,751,184–74,100,813) × 1	1.35 Mb	+	+	+	−	+	+
9	M	13	CMA	Del (7q11.23) (72,073,782–76,132,541) × 1	4.06 Mb	+	−	+	−	−	−
Howald et al. [13]	M	36	PSQ	NR	NR	−	+	+	NR	−	+
Tassabehji et al. [30]	F	92	FISH	NR	NR	+	+	+	NR	−	+
Gagliardi et al. [31]	M	66	FISH	NR	NR	+	+	+	NR	NR	+
Korenberg et al. [32]	M	24	FISH	NR	NR	+	+	+	−	NR	+

*CMA* chromosomal microarray analysis, *WBS* Williams–Beuren syndrome, *PSQ* paralogous sequence quantification, *FISH* fluorescent in situ hybridization, *NR* not reported, Present (+) and not present (−)

### Growth status

By measuring the height and weight of patients, the Z score analysis method and WHO Anthro software were used to calculate the Z score, as shown in Additional file 1: Table S1. According to the WHO growth standard reference, only three patients (No. 3, No. 6, and No. 7) showed normal growth and development. Patients No. 3 and No. 5 were diagnosed as low-birth-weight children because their birth weight (2.1 kg for No. 3 and 1.88 kg for No. 5) was below 2.5 kg. All patients’ gestational age was older than 37 weeks (37–40 weeks).

### Cardiovascular phenotypes

Except for patient No. 9, all patients showed cardiovascular abnormalities. Among them, SVAS and PS were the most common cardiovascular diseases. Patient No. 3 had undergone surgical correction because of severe supravalvular pulmonary stenosis at 5 months. The details of the cardiovascular diseases are shown in Additional file 2: Table S2.

### Neuropsychological testing

According to the information provided by the patient’s parents, all patients, except for patient No. 7, suffered from significant delays in developmental milestones, such as standing and walking independently and saying their first word and their first sentence.

According to their parents’ recollection, most patients could walk without support at approximately 16–18 months, and they could only say a few simple words after 20 months, such as father and mother. “They were not able to say a complete sentence consciously until 3 years of age”. However, the development of patient No. 7 was normal. He could sit at 7 months, walk alone at 12 months, and speak short sentences with 6–10 words at 2 years of age.

The patients’ GDS scores are shown in Table 2. The results of 20 patients with typical deletions of WBS in the same age group are also shown for comparison. All patient test data were obtained before any medical intervention. The results of the assessment were almost consistent with the information provided by parents. Except for patient No. 7, the total average GDS scores of all patients remained below 85.

**Table 2:** Comparison of GDS between patients with typical deletions and those with atypical deletions

Case no	GDS, DQ					
	Gross motor	Fine motor	Adaptive behavior	Language	Social behavior	Total average
1	53	67	70	54	78	64
2	60	57	66	61	58	60
3	75	75	69	60	55	67
4	65	60	69	60	55	62
5	58	47	53	50	60	54
6	76	58	55	57	67	63
7	108	90	83	83	93	91
8	70	40	55	39	50	51
9	47	33	45	34	40	40
Typical deletions*	61 ± 14.5	60 ± 20	59 ± 14	54 ± 18	58 ± 18.5	60 ± 15

*GDS* Gesell development scale, *DQ* developmental quotient

*Mean ± SD

### Facial features

Most of the nine patients with atypical deletions had distinct faces (see Additional file 3: Table S3), similar to those with typical deletions. However, patients No. 2 and No. 9 did not have specific facial features related to the syndrome. For example, patient No. 2 looked the same as ordinary people except for swelled tissue around the orbit. Unfortunately, the patients’ parents did not agree to publish photos.

### Other clinical symptoms

Five patients (No. 1, No. 3, No. 5, No. 6, and No. 7) developed inguinal hernia within 6 months of birth. Except for patient No. 1, who required surgical intervention, all patients recovered. Patient No. 6 was diagnosed with subclinical hypothyroidism because of elevated thyroid stimulating hormone (TSH) levels (7.519 μIU/mL, normal range, 0.560–5.910 μIU/mL) with normal T3 and T4 levels. The blood calcium level of patient No. 8 was 2.81 mmol/L (normal range, 2.25–2.75 mmol/L); thus, this patient No. 8 diagnosed with mild hypercalcemia.

## Discussion

Nine WBS cases of atypical deletions were identified in our study. One patient with normal neurodevelopment had a gene deletion on the distal side of the chromosome region of Williams–Beuren syndrome that included *GTF2I* and *GTF2IRD1* genes. The other patients retained these genes but showed abnormal neurodevelopment. These results indicate that if only the genes on the distal side of the WBSCR are deleted, especially the *GTF2I* and *GTF2IRD1* genes, the effects are not sufficient to cause a neurodevelopmental delay in patients with WBS. This seems to contrast with previous reports [6, 7], which showed that the genes on the distal side of the WBSCR play a major role in the WBS phenotype. Moreover, based on patient No. 6, the deletions of *BAZ1B* and *FZD9* located on the proximal side of the WBSCR, as well as *STX1A* deletion may also contribute to the typical neurocognitive phenotype of WBS. This suggests that the deletion of genes on the proximal side of the WBSCR also exerts an equally important effect on the phenotype of WBS. Furthermore, it can be inferred that the main target gene that causes growth retardation in patients with WBS is *WBSCR22*. WBS patients with smaller and larger deletions may not have a typical clinical phenotype, which detrimentally affects the precise diagnosis and treatment by clinicians. At this time, complete genetic testing is particularly important. This is important not only for the purpose of genetic counseling but also can establish a targeted follow-up. Moreover, patients with atypical deletion play an important role in the study of gene functions.

Nine (8%) out of 111 Chinese patients with WBS had atypical deletions, which exceeded the ratio of 2–5% that was reported in previous literature. Possible reasons include the advancement of genetic testing technology, which has increased the detection rate of atypical deletions. A further reason is the deepening of people’s understanding of the disease, which has led to the diagnosis of many WBS patients with nonclassical clinical phenotypes. For patients with suspected WBS, especially those with nonclassical clinical phenotypes, CMA or next-generation sequencing (NGS, i.e., high-resolution molecular testing, such as whole exome sequencing (WES), whole genome sequencing (WGS) and targeted region sequencing (TRS) [20, 21]) is recommended to obtain more accurate and complete genetic information.

**Fig. 2:**
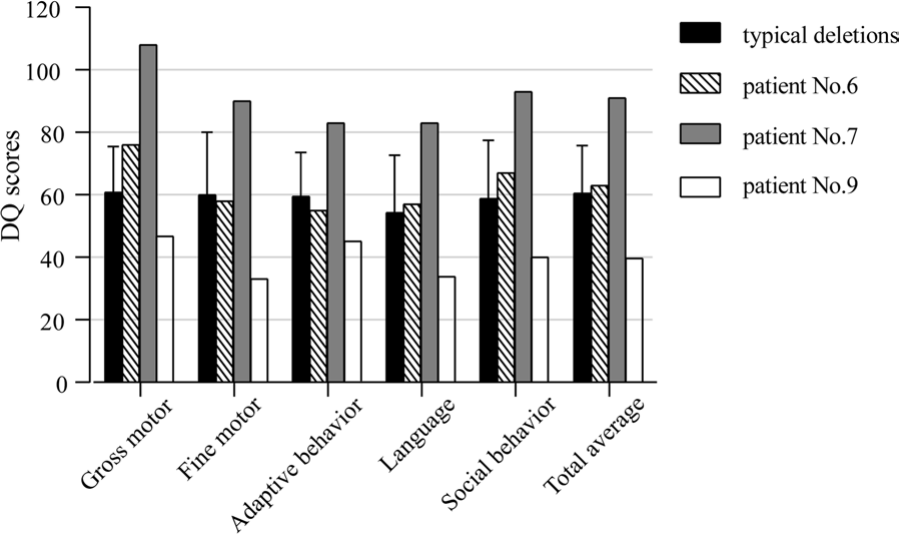
Neurocognitive development assessment scores of patients No. 6, No. 7, and No. 9 and 20 WBS patients with typical deletions. The neurocognitive development of patient No. 7 is normal, while patient No. 6 and patients with typical deletions have developmental limitations. In addition, patient No. 9 had the worst neurocognitive development. *DQ* development quotient

Neurologic and intellectual disability is one of the most important and common features in patients with WBS [1, 22, 23]. This study used GDS to assess patients’ neurodevelopmental status; this metric includes the five main functional areas of the human body. All patients with atypical deletions (except patient No. 7) showed mild to moderate intellectual disability similar to those with typical deletions. The age and size of chromosome deletions were similar between patients No. 6 and No. 7, but their GDS results were significantly different (Fig. 2). This suggests that the differences between them may be related to the different positions of chromosome breakage and gene deletions.

*GTF2I* and *GTF2IRD1* belong to the same transcription factor family. They interact with a variety of proteins and DNA to influence neurophysiology and developmental processes [24]. Previously, the heterozygous deletion of the *GTF2I* and *GTF2IRD1* genes have been reported as the main cause of neurocognitive characteristics, special facial features, and motor dysfunction in WBS patients [7, 25, 26]. However, these two genes were deleted in patient No. 7 of this study, but this patient showed normal neurological development. In contrast, in patient No. 6 the deleted genes were primarily located on the centromere side of the WBSCR (from *FKBP6* to *ELN*), and this patient showed typical WBS cognitive characteristics.

An in-depth study of the molecular and phenotypic characteristics of patient No. 7 showed that the language and adaptive development of the patient were in a marginal state. This may be because the genes *GTF2I* and *GTF2IRD1* mainly affect the neurodevelopment related to these two functional regions [27, 28], but they are not sufficient to cause the intellectual disability symptoms typical for WBS patients. However, it cannot be ruled out that CNV size-related position effects, variants in the allele not deleted, epigenetic mechanisms, regulatory sequences, or other factors may affect a patient’s phenotype [29]. Analysis of the molecular and phenotypic relationship between patient No. 6 and previous case reports [13, 30–32] with similar deletion positions and intellectual disability showed that the genes on the proximal side of the WBSCR also play an important role in the phenotype of patients with WBS. The *BAZ1B*, *FZD9*, and *STX1A* genes are particularly important in this regard, according to previous studies [9, 33–37]. The *BAZ1B* gene, also known as Williams syndrome transcription factor (WSTF), plays an important role in the differentiation and migration of nerve cells. It also participates in the neural crest specific transcription loop and remote regulation [33]. *Wnt* signaling plays an important role in the regulation of the balance between the proliferation and differentiation of neural progenitor cells. Inhibition or overexpression of *Wnt* signaling function can lead to a decrease in or proliferation of neural progenitor cells, respectively. The *BAZ1B* gene is enriched in the *Wnt* signal transduction pathway. Thus this pathway is activated because of the deletion of this gene in WBS patients [38, 39]. This affects the proliferation and differentiation of nerve cells in patients with corresponding neurocognitive phenotypes. Studies [33, 40] have shown that the *BAZ1B* gene is associated with the facial features and behavioral phenotypes of WBS patients. Furthermore, a recent study [41] suggested that through the PTEN-mediated pathway, the deletion of *BAZ1B* gene heterozygosity reduces both the viability and survival of thyroid cells, thereby causing hypothyroidism in patients with WBS. Moreover, the *BAZ1B* gene is also involved in the development of sperm, and its deletion may be one of the influencing factors of infertility in WBS patients. Knockout of the *BAZ1B* gene can cause changes in the chromosome aggregation phase of cells and errors in this process. This may lead to delays in the prophase of mitosis, which may affect sperm development [42]. Therefore, its deletion may be one of the influencing factors of infertility in WBS patients. It should be noted that the *FZD9* gene also plays a role in the *Wnt* signaling pathway [43]. By increasing the doubling time and apoptosis of nerve cells, the deletion of the *FZD9* gene can affect the development of the nervous system and cause cognitive impairment [9]. Previous studies [44] have shown that *FZD9* is highly expressed in the hippocampus and is involved in cognition and memory. Endogenic expression of *FZD9* can promote synaptic formation in hippocampal neurons through the *Wnt* pathway [9, 34]. In mice, the deletion of the *FZD9* gene increased apoptosis in developing dentate gyrus cells and compensated for the proliferation of the number of dentate gyrus division precursors [44]. Mice with heterozygous mutations in the *FZD9* gene had severe deficits in visuospatial learning and memory. This evidence suggests that the *FZD9* gene is an important factor in hippocampal development, and its heterozygosity deletion may be one of the contributing factors to neurodevelopment and behavioral phenotypes in patients with WBS. The *STX1A* gene encodes a neuronal soluble N-ethylmaleimide-sensitive fusion attachment protein receptor, which promotes nerve function in the central nervous system by regulating the release of transmitters [45]. Recent studies have shown that mutations or deletions of *STX1A* are related to human neuropsychological diseases, such as autism spectrum disorder and attention deficit hyperactivity disorder [46, 47]. *STX1A* is expressed primarily in the brain regions involved in learning, memory, and fear (the cortex, hippocampus, and amygdala, respectively) [48]. By knocking out the *STX1A* gene, the synaptic transmission of hippocampal neurons in mice was normal. However, the long-term enhancement of hippocampal neurons was impaired and conditioned fear memory disappeared [45]. Combined with these results, *STX1A* may be an interesting candidate gene for learning and memory deficits in WBS individuals. However, the underlying mechanism of this deficiency and its contribution to the neurocognitive symptoms of WBS remain unclear. These studies will enable a deeper understanding of the genotype–phenotype correlation in WBS microdeletions, and help to understand the molecular mechanisms of diseases and the human social brain.

Previous reports have shown that clinical symptoms are also affected by the size of the deletion [6, 49]. In comparison to other patients with atypical deletions, the chromosome deletions of patient No. 9 were larger and the neurodevelopmental delay was more severe (Fig. 2), which is consistent with previous reports [49, 50]. This suggests that the deletion of the extension genes *HIP1* and *YWHAG* on the distal side of the WBSCR inhibits neurodevelopment [51]. The data of this study also showed that patients with large deletions (such as patients No. 2 and No. 9) did not have classical facial features, which may be related to the size and position of the deleted fragments. Thus, focus should be given to the diagnosis of patients with nonclassical atypical WBS.

Growth restriction is another characteristic of WBS patients [4]. The *WBSCR22* gene encodes a putative methyltransferase protein that is strongly expressed in the heart, skeletal muscle, and kidney. Its heterozygous deletion may lead to growth retardation, myopathy, or premature aging [52]. In this study, only patient No. 7 retained the *WBSCR22* gene and showed normal physical development. However, seven of the eight (87.5%) patients with deletion of this gene showed growth restriction, of which patient No. 3 had low birth weight. This suggests that this effect may be caused by the deletion of the *WBSCR22* gene. However, growth is affected by many factors, such as diet, endocrine function, and the environment. Therefore, more research is needed to verify the effect of the *WBSCR22* gene on the growth and development of WBS patients.

The *ELN* gene encodes elastic fibers, which are essential elements of the extracellular matrix. Heterozygous deletion of the *ELN* gene is the main cause of cardiovascular abnormalities in WBS patients, especially SVAS and PS [5]. All patients in this study had a heterozygous deletion of the *ELN* gene, and all patients (except for patient No. 9) developed cardiovascular disease, which is consistent with previous reports [53]. The possible reason why patient No. 9 has not yet developed a relevant cardiovascular phenotype may be the young age of the patient. Alternatively, deletion of genes outside the WBSCR region may also be related to the cardiovascular phenotype of this patient.

## Conclusions

The *BAZ1B*, *FZD9*, and *STX1A* genes may play an important role in the neurodevelopment of patients with WBS. Furthermore, deletion of the *WBSCR22* gene may be the main cause of physical growth restriction in WBS patients. Identifying patients with an atypical deletion of WBS has important clinical and scientific significance. To study the contribution of each gene to the patient’s phenotype, more subjects with atypical deletions are needed to better understand their molecular-phenotype relationship. Animal experiments can then be used to study and verify the function of relevant genes.

## Supplementary Information

**Additional file 1: Table S1.** Evaluation results of the physical development of patients with atypical deletion by the Z-score method.

**Additional file 2: Table S2.** Type of cardiovascular diseases in nine WBS patients with atypical deletion.

**Additional file 3: Table S3.** Facial features of nine WBS patients with atypical deletion.

## Data Availability

To ensure patient confidentiality, data containing potentially identifiable information was not shared. The datasets generated during and/or analysed during the current study are not publicly available due to these data being derived from clinical genetic testing; however, deidentified data may be available from the corresponding author on reasonable request.
