# Supplementary material for: Atypical deletion of Williams–Beuren syndrome reveals the mechanism of neurodevelopmental disorders
Source: BMC Med Genomics. 2022 Apr 4;15:79. doi: 10.1186/s12920-022-01227-7 (PMC8981662; doi:10.1186/s12920-022-01227-7)
Supplement: Supplementary file 1 — Additional file 1: Table S1. Evaluation results of the physical development of patients with atypical deletion by the Z-score method. [file 12920_2022_1227_MOESM1_ESM.docx]

Table S1 Evaluation results of the physical development of patients with atypical deletion by the Z-score method

| Case No. | WAZ | HAZ | BAZ | Growth abnormalities |
| --- | --- | --- | --- | --- |
| 1 | -1.69 | 0.43 | -2.88 | + |
| 2 | -2.58 | -3.71 | 0.2 | + |
| 3 | 0.52 | -0.31 | 0.98 | - |
| 4 | -2.14 | -1.98 | -1.35 | + |
| 5 | -4.02 | -4.8 | -1.69 | + |
| 6 | -1.26 | -0.73 | -1.27 | - |
| 7 | 0.28 | 0.32 | 0.08 | - |
| 8 | -1.76 | -2.29 | -0.53 | + |
| 9 | -2.09 | -1.89 | -1.37 | + |

BAZ, BMI-for-age z-score; HAZ, Height-for-age z-score; WAZ, Weight-for-age z-score.

Present (+) and not present (-)
