# Supplementary material for: Atypical deletion of Williams–Beuren syndrome reveals the mechanism of neurodevelopmental disorders
Source: BMC Med Genomics. 2022 Apr 4;15:79. doi: 10.1186/s12920-022-01227-7 (PMC8981662; doi:10.1186/s12920-022-01227-7)
Supplement: Supplementary file 2 — Additional file 2: Table S2. Type of cardiovascular diseases in nine WBS patients with atypical deletion. [file 12920_2022_1227_MOESM2_ESM.docx]

Table S2 Type of cardiovascular diseases in nine WBS patients with atypical deletion

| Case No. | Cardiovascular diseases | | | | | | | |
| --- | --- | --- | --- | --- | --- | --- | --- | --- |
|  | SVAS | PS | CoA | ASD | SVPS | PDA | PVS | MVP |
| 1 | + | + | + | - | + | - | - | + |
| 2 | + | + | + | + | - | - | - | - |
| 3 | + | + | - | + | + | - | + | - |
| 4 | + | - | + | - | - | - | - | - |
| 5 | + | + | - | + | - | + | + | - |
| 6 | + | - | - | - | - | - | - | - |
| 7 | + | + | - | - | - | - | - | - |
| 8 | + | + | - | - | - | - | - | - |
| 9 | - | - | - | - | - | - | - | - |

ASD, atrial septal defects; CoA, coarctation of the aorta; MVP, mitral-valve prolapse; SVAS, supravalvular aortic stenosis; PDA, patent ductus arteriosus; PS, pulmonary stenosis; PVS, pulmonary valve stenosis; SVPS, supravalvular pulmonary stenosis.

Present (+) and not present (-).
