# Supplementary material for: Atypical deletion of Williams–Beuren syndrome reveals the mechanism of neurodevelopmental disorders
Source: BMC Med Genomics. 2022 Apr 4;15:79. doi: 10.1186/s12920-022-01227-7 (PMC8981662; doi:10.1186/s12920-022-01227-7)
Supplement: Supplementary file 3 — Additional file 3: Table S3. Facial features of nine WBS patients with atypical deletion. [file 12920_2022_1227_MOESM3_ESM.docx]

Table S3 Facial features of nine WBS patients with atypical deletion

| Facial features | Typical deletion | Case No. | | | | | | | | |
| --- | --- | --- | --- | --- | --- | --- | --- | --- | --- | --- |
|  |  | 1 | 2 | 3 | 4 | 5 | 6 | 7 | 8 | 9 |
| Flat nasal bridge | + | + | - | + | + | + | + | + | + | + |
| Short upturned nose | + | + | - | + | + | - | + | - | + | - |
| Periorbital puffiness | + | + | + | + | + | + | + | + | + | - |
| Long philtrum | + | + | - | + | + | + | + | + | + | + |
| Delicate chin | + | + | - | + | + | - | + | + | + | - |
| Full lips | + | - | + | - | - | - | + | - | - | - |
| Full cheeks | + | + | + | + | + | + | + | + | + | - |
| Wide mouth | + | + | - | + | - | + | + | - | + | - |

Present (+) and not present (-).
